# Newly detected DNA viruses in juvenile nasopharyngeal angiofibroma (JNA) and oral and oropharyngeal squamous cell carcinoma (OSCC/OPSCC)

**DOI:** 10.1007/s00405-018-5250-7

**Published:** 2018-12-21

**Authors:** Amy Dickinson, Man Xu, Suvi Silén, Yilin Wang, Yu Fu, Mohammadreza Sadeghi, Mari Toppinen, Timo Carpén, Klaus Hedman, Antti Mäkitie, Maria Söderlund-Venermo

**Affiliations:** 1 Department of Otorhinolaryngology, Head and Neck Surgery, University of Helsinki, Helsinki University Hospital, Helsinki, Finland; 2 Department of Biosciences and Nutrition, Karolinska Institute, Stockholm, Sweden; 3 Department of Virology, University of Helsinki, Helsinki, Finland; 4 Helsinki University Hospital, Helsinki, Finland; 5 Division of Ear, Nose and Throat Diseases, Department of Clinical Sciences, Intervention and Technology, Karolinska Institute, Karolinska University Hospital, Stockholm, Sweden; 6 Department of Virology, University of Turku, Turku, Finland

**Keywords:** Juvenile nasopharyngeal angiofibroma, Head and neck cancer, Protoparvovirus, Human parvovirus B19, Bocavirus, Polyomavirus, Merkel cell virus

## Abstract

**Purpose:**

Approximately 20% of cancers are estimated to have a viral etiology. We aimed to investigate whether DNA of 8 human parvoviruses [bocavirus 1–4 (HBoV1–4), parvovirus B19 (B19V), protoparvoviruses (bufa-, tusa-, and cutavirus)] and 13 human polyomaviruses (HPyV) can be detected in oropharyngeal and oral cavity squamous cell carcinoma (OPSCC/OSCC), and in juvenile nasopharyngeal angiofibroma (JNA) tissue samples.

**Methods:**

Fresh samples of seven JNA tissues and ten paired tissues of OSCC/OPSCC tumor and adjacent healthy tissues were collected. DNA extraction and real-time PCRs were performed to detect HBoV1-4, B19V, bufa- tusa- and cutavirus, and HPyV genomes.

**Results:**

JNA specimens were negative for all parvoviruses tested, whereas one JNA sample was Merkel cell polyomavirus (MCPyV) DNA positive. The OSCC/OPSCC samples were negative for the human protoparvoviruses, HBoV1-4, and all human polyomaviruses, except for one patient that was MCPyV DNA positive in both healthy and tumor tissues. Seven OSCC/OPSCC patients were positive for B19V DNA, three of them in both healthy and cancerous tissues and three in only healthy tissues. Three of the B19V DNA-positive patients harbored viral genotype 1, three genotype 2, and one genotype 3B.

**Conclusions:**

These are the first reports of MCPyV and B19V DNA being detected in JNA and OPSCC. The significance of viral DNA positivity is unclear. B19V DNA is known to remain in the tissues lifelong, however, it is of interest that there are some patients with B19 DNA in healthy tissue, but not in the corresponding cancer tissue.

## Introduction

An estimated 15–20% of cancers are a consequence of viral infection, such as human papillomaviruses (HPV) in cervical carcinomas and oropharyngeal squamous cell carcinoma (OPSCC) and hepatitis B and C in liver cancers. However, many other known and unknown viruses may trigger carcinogenesis through modification of apoptosis, cell division and differentiation, as well as signaling cascades [1].

Head and neck squamous cell carcinoma (HNSCC) has a worldwide annual incidence of approximately 600,000 cases [2]. HPV and EBV are widely known etiological factors for oropharyngeal and nasopharyngeal carcinoma [1], respectively. In Western countries in the last few decades, the incidence of some HNSCCs has been decreasing, along with a decrease in risk factors such as smoking. Despite this, the incidence of OPSCC has been increasing, likely in conjunction with HPV. However, not all of those infected with high-risk HPV develop squamous cell carcinoma [3], therefore, it is possible that other viruses act as co-factors. Moreover, oral cavity squamous cell carcinoma (OSCC) incidence has been rising, especially in < 45-year-old individuals and patients without typical risk factors or HPV [2]. Their etiology needs to be better understood: specifically, whether there is an undiscovered viral component.

Juvenile nasopharyngeal angiofibroma (JNA) is a rare, highly vascular tumor of the nasopharynx, almost exclusively in young males. It is the most common benign nasopharyngeal neoplasm, comprising 0.5% of all head and neck tumors. Despite being histologically benign, it is locally destructive, and can lead to bony remodeling and even intracranial extension [4]. Its etiology is poorly understood. However, our preliminary results from previous studies have pointed to the possibility of a viral etiology. First, there was upregulation of immunological proteins such as TNF alpha in a higher stage JNA sample [4]. Additionally, lymphocytes and mast cells, usually present in chronic infection, were seen within two JNA tissue samples, sparking work to look at toll-like receptors (TLR) within JNA samples. The intracellular TLRs 3, 7 and 9, thought to recognize viral nucleic acids, have been found within the JNA tissue [5]. Therefore, it seemed relevant to investigate a viral etiology for JNA. Furthermore, HPV has been discovered in all samples in a small JNA cohort [6].

In this study, we aim to investigate the presence of novel viruses in both JNA and OPSCC/OSCC, of which there is little or no documentation in the literature, given a precedent of viral illness’ role in cancer development. The viruses included in this study are 8 human parvoviruses: [parvovirus B19 (B19V), bocaviruses (HBoV) 1–4], bufavirus (BuV), tusavirus (TuV), and cutavirus (CuV); as well as 13 human polyomaviruses (HPyVs): BK, JC, KI, WU, trichodysplasia spinulosa (TS), Merkel cell virus (MC), Malawi (MW), St Louis (STL), New Jersey, and human polyomaviruses 6, 7, 9, 12 and 13.

## Materials and methods

### Clinical specimens

The clinical material comprised fresh JNA tissues from seven Finnish males (age range 17–33 years; mean 22.7, median 22), as well as paired samples of ten tumor and eight healthy mucosa from ten patients with OSCC/OPSCC (age range 31–86 years; mean 61.2, median 59). The healthy tissues were taken from healthy buccal mucosa on the contralateral side. The patients with OSCC/OPSCC encompassed seven males and three females. Nine were Finnish, whereas one male was Bulgarian, and had moved to Finland. Tumor information can be found in Table 1. Clinical TNM stage was used if the patient did not have operative treatment and pathological TNM stage used if the patient underwent an operation.

**Table 1:** Demographics and details of oral and oropharyngeal tumor specimens, along with parvovirus B19 and Merkel cell polyomavirus status. The PCR assays for the remaining viruses tested were all negative

Gender	Age, years	Tumor location	TNM (all M0)	TNM stage	Tumor B19V status (DNA copies per 10^6^ cells)	Healthy sample B19V status (DNA copies per 10^6^ cells)	B19V genotype	MCPyV presence
OPSCC								
Male	58	Base of tongue	T4aN0	IVa	+ (41)	+ (251)	1	+
Male	52	Tonsil	pT2pN2a	IVa	+ (6)	+ (158)	1	−
Female	66	Soft palate	T1N2c	IVa	+ (2)	+ (12)	2	−
Male	52	Soft palate	pT2bpN2a	IVa	+ (131)	+ (301)	3B	−
Male	85	Tonsil	T2N2	IVa	−	N/A	N/A	−
Male	46	Tonsil	T2N2b	IVa	−	+ (11)	1	−
Male	31	Tonsil	T2N2b	IVa	−	−	N/A	−
Male	76	Tonsil	pT2bpN0	II	−	N/A	N/A	−
OSCC								
Female	86	Lateral tongue	T3N1	IV	−	+ (125)	2	−
Female	60	Cheek mucosa	pT2pN2b	IV	−	+ (20)	2	−

*B19V* parvovirus B19, *MCPyV* merkel cell virus, *TNM* tumor node metastasis, + positive, − negative, *N/A* not applicable

All tissues were freshly frozen directly after surgical resection in the operating theatre. DNA from frozen biopsies were extracted using the QIAamp DNA mini kit (Qiagen). All procedures were carried out according to the manufacturers’ protocols. The DNA preps were eluted in 100 µl water.

### Viral DNA assays

All the real-time qPCR assays were performed with Stratagene Mx3005P (Agilent Technologies, USA). Plasmids containing either full-length virus genomes or corresponding PCR amplicons were used as amplification standards in each PCR.

An HBoV1-4 multiplex qPCR assay was applied to detect HBoV1-4 genomes. The PCR amplifies the HBoV genome from the left-hand non-coding region to the beginning of the *NS1* gene. The plasmids and qPCR protocol are described elsewhere [7].

A Pan-B19 qPCR targeting the *NS1* gene of the viral genome was used to detect and amplify all three B19V genotypes, as described [8]. To confirm the B19V Gt3B-positive samples, a nested PCR was performed to amplify a 1064-bp fragment of the *VP1* gene, as described [9]. The alignment of B19V Gt3B-positive samples was done with MUSCLE and the phylogenetic analysis was performed by constructing maximum likelihood trees using PhyML program with HKY85 nucleotide substitution model (Phylogeny.fr).

A multiplex real-time qPCR was used for the detection of the three human protoparvoviruses, with primers located in the *NS1* region of BuV and the *VP2* region of TuV and CuV, as described [10].

DNAs of 13 HPyVs were detected with a Luminex-based multiplex assay by targeting the respective *VP1* genes of HPyVs, as described [11].

All DNA extracts were subjected to the reference gene, *RNase P*, qPCR for quality control and cell-count quantification, as described [8].

Ethics/Permissions: This research is part of the “Medical significance of new DNA viruses” study, approved by the Ethics Committee of Helsinki and Uusimaa Hospital District and the National Supervisory Authority for Welfare and Health (Valvira). Written consent was obtained from all individual participants included in the study for their tissue samples to be collected and used in the research.

## Results

### JNA samples

All JNA tissue samples were DNA negative for all parvoviruses (BuV, TuV, CuV, HBoV1-4 and B19V), as well as for all human polyomaviruses, except one sample, from a 33-year-old patient, which was positive for MCPyV DNA.

### Head and neck cancer specimens

All samples were negative for BuV, TuV, CuV and HBoV1-4. In one patient with a base of tongue cancer, both the tumor sample and the matched healthy mucosa were positive for MCPyV; the remaining samples were negative for any HPyV DNA. However, 11/18 samples from 7 out of 10 patients were positive for B19V DNA. Four of these 7 patients had B19V DNA in both cancer and healthy tissues, and three in only the healthy tissues but not in the cancer. The four tumor samples that were positive for B19V DNA were all TNM stage IVa. They were of varying tumor types: two of the soft palate, one of the base of tongue, one of the tonsil. Of the B19V-DNAs detected, three were of genotype 1, three of genotype 2, and one of genotype 3B. The latter was from a 52-year-old male patient who was originally from Bulgaria. The viral load was low among all B19V DNA-positive samples (mean 96.2, median 41, range 2–301 copies per one million human cells). Table 1 shows the details of the tumors and patient demographics, along with B19V viral counts and genotypes, and HPyV presence.

## Discussion

This panel of viruses was selected for varying reasons. Polyomaviruses are known to cause tumors in animals, and to date MCPyV, discovered in 2008, has been detected in 70–80% of Merkel cell carcinomas in humans [12]. HBoV1-4, belonging to the *Parvoviridae* family, are mainly present in respiratory or enteric infections, however, in two studies HBoV1 was found in 20% of colorectal and 18% of lung cancers [13, 14]. BuV, TuV and CuV are protoparvoviruses discovered in pediatric diarrheal stool samples and CuV was additionally found in skin lesions of cutaneous T-cell lymphoma (CTCL) [15, 16]. It was later detected in both healthy and malignant skin of CTCL and immunosuppressed organ transplant patients, but not in skin of healthy adults [10]. Parvoviruses in general are not considered directly oncogenic like the polyomaviruses. However, prolonged antigen stimulation by persistent viruses could induce chronic inflammatory responses that together with other molecular events would lead to cell mutations and cell proliferation. Alternatively, parvoviruses could prefer rapidly dividing cancer cells for their replication and lyse the target cells, thus shrinking or even curing the tumor. It is imperative to study if and how viruses like these affect tumor onset, progression and outcome.

Previous research detecting viruses within JNA have studied HPV, human herpesvirus-8 (HHV-8) and EBV: in a small sampling of 6 patients, all were positive for HPV [6], and in another study, all 15 JNA tissue samples were negative for HHV-8 and EBV [17]. Our results are the first published on B19V, HBoV1-4, BuV, TuV, CuV, and 13 HPyVs within JNA samples, showing negative findings in all patients except one, whose JNA was positive for MCPyV DNA, a known oncogenic virus found in the majority of Merkel cell carcinomas [12]. This is the first documented case of MCPyV DNA within JNA tissue. However, the MCPyV is a relatively ubiquitous virus, with a seroprevalence in adults of 50–95%. The majority of adults further shed MCPyV from their skin [12]. Thus, this finding in the oldest patient of the cohort could be a reflection of his previous encounter with the virus. Although exceedingly rarely, Merkel cell carcinomas have been reported within the nasal vestibule [18]; therefore, it is conceivable that MCPyV may also be present in the sinonasal passages.

In one recent HNSCC study, viral signatures were detected from 100 OSCC/OPSCC samples [19]. HPV 16 was found in 98% of the OSCC/OPSCCs but not in controls, and reo-, herpes-, pox-, orthomyxo-, retro- and polyomaviruses were found with a hybridization signal level 2–3 logs higher than in the controls, all showing statistically significant differences. The presence of EBV in this study was statistically associated with OSCC [20].

A recent study of HPyV DNA detection in 82 HNSCC samples by Poluschkin et al. revealed HPyV positivity in 52% (83% of 29 OSCCs and 0% of 23 OPSCC) [21]. In OSCC, JCPyV was present in 59%, SVPyV in 21% and BKPyV in 3% of the samples. Interestingly, in 86.2% of JCPyV-positive HNSCC samples, there was HPV co-infection. In our study, one out of eight OPSCC samples was positive for HPyV (MCPyV), and both OSCC samples were negative for HPyV. The discrepancy between this study and our study, both on Finnish patients, could be due to local variance, as the patient cohorts were from different regions, small sample size, or perhaps due to different methods used in the detection of the viruses.

Parvovirus B19 can persist lifelong in human body tissues [22]. Of the patients with B19V DNA-positive tumor samples, all harboring genotype 1 were under 60 years of age, whereas those with genotype 2 were over 60 years of age, correlating with the fact that B19V genotype 2 widely circulated around 50 years ago, but then disappeared from circulation around 1970 [23]. One patient was found to harbor, in both healthy and cancerous tissue, DNA of B19V genotype 3, which is extremely rare in Finland—indeed, this patient had travelled from Bulgaria. Hitherto, only two subjects have been found positive for genotype 3 in this region, and they were soldiers from the Soviet army killed during World War II [24]. In the current study, B19V DNA was present in 11/18 samples of 7/10 patients, however, in three of the patients the cancer tissue was negative for B19V while the paired healthy tissue was positive. The role of B19V existing in the control tissues more than the cancer tissues of the same patients is unclear at this point.

For the recently discovered boca-, bufa-, tusa-, and cutaviruses, this is the first study of their presence in OSCC/OPSCC, however, CuV has been detected in skin lesions of CTCL, melanoma, basal cell and squamous cell carcinomas [10, 16, 25].

In conclusion, PCRs for B19V and the emerging parvoviruses HBoV1-4, BuV, TuV, and CuV, as well as for 12 of 13 HPyVs were negative in the studied JNA samples, whereas MCPyV was detected in one. This was the first report of MCPyV DNA being found in JNA. The OSCC/OPSCC samples were DNA positive only for MCPyV in one patient, and B19V DNA in 11/18 samples in 70% of the patients, however, the significance of this viral presence remains unclear.
